# Supplementary material for: The Oncology Biomarker Discovery framework reveals cetuximab and bevacizumab response patterns in metastatic colorectal cancer
Source: Nat Commun. 2023 Sep 4;14:5391. doi: 10.1038/s41467-023-41011-4 (PMC10477267; doi:10.1038/s41467-023-41011-4)
Supplement: Supplementary file 1 — Supplementary Information [file 41467_2023_41011_MOESM1_ESM.pdf]

Supplementary information for

The Oncology Biomarker Discovery framework reveals cetuximab and bevacizumab response patterns in metastatic colorectal cancer

Ohnmacht AJ, Stahler A, Stintzing S, Modest DP, Holch JW, Westphalen CB, Hölzel L, Schübel MK, Galhoz A, Farnoud A, Ud-Dean M, Vehling-Kaiser U, Decker T, Moehler M, Heinig M, Heinemann V, Menden MP

Corresponding:
Menden MP. (michael.menden@helmholtz-munich.de),
Heinemann V. (volker.heinemann@med.uni-muenchen.de)

This PDF file includes:

Supplementary Figure 1 to 16
Supplementary Table 1

Other supplementary information for this manuscript include:

Supplementary Data 1 to 4

Method abbreviation	Name	Implementation	Modelling type	Control of false positive rate	Output type	Found subgroups (OS)	Hazard ratio on OS for subgroup not in standard treatment and discovered by method [p-value; n = amount of patients]	Execution time [mins]
OncoBird	Oncology Biomarker Discovery	R package 'OncoBird'	stratified linear regression with interactions	Benjamini-Hochberg correction; permutations and bootstrapping for treatment effect correction	subgroups with interaction effects in predefined subgroups	*KRAS* mutations in CMS4; *KRAS/NRAS/SRC/BRAF* alterations in CMS4; *KRAS/NRAS/SRC* alterations in CMS4; *TOP1* amplification in CMS2; *ARFRP1* amplifications in CMS2	HR = 0.57 (p = 0.16, n = 29)	2.2
VT	Virtual twins	R package 'randomforestSRC'	random forests and regression tree	cross-validation	decision tree on biomarkers from global outcomes	*KRAS/NRAS/BRAF* alterations not in CMS2; CMS3	HR = 0.65 (p = 0.17, n = 48)	1.2
MOB	Model-based recursive partitioning	R package 'model4you'	recursive chi-squared independence tests	Bonferroni-correction	decision tree on recursive treatment effects	*KRAS/NRAS/BRAF/IRS2/NF1* alterations	HR = 0.82 (p = 0.62, n = 28)	0.1
OWE	Outcome weighting estimation	R package 'personalized'	outcome weighting or A-learning	cross-validation; bootstrapping	subgroups with interaction effects	*NRAS* mutations in left-sided; *SOX9* mutations in left-sided	HR = 0.87 (p = 0.44, n = 150)	14.7
CRF	Causal random forests	R package 'grf'	generalised random forests	honest trees	variable importance scores for treatment effects	*KRAS/NRAS/BRAF/IRS2/NF1* alterations; *KRAS/NRAS/BRAF* alterations; *ATM/TOP1* alterations	HR = 0.63 (p = 0.12, n = 50)	0.1
POL	Policy learning	R package 'policytree'	semi-parametrically efficient estimation	honest trees	decision tree on found subgroups	*KRAS/NRAS/BRAF* alterations; *PIK3CA/PTEN/GNAS/ERBB2* alterations in left-sided; *ARID1A/SMAD2/CUL4A* alterations	HR = 0.76 (p = 0.18, n = 117)	0.1
GUIDE	Generalized, unbiased, Interaction detection and estimation	Binary executable *https://pages.cs.wisc.edu/~loh/guide.html*	interaction tests	cross-validation and bootstrapping for treatment effect correction	subgroups with interaction effects	*KRAS/NRAS/BRAF* alterations; CMS3	HR = 0.76 (p = 0.30, n = 60)	0.4
PRISM	Patient response identifiers for Stratified Medicine	R package 'StratifiedMedicine'	virtual twins or model-based partitioning with post parameter estimation	bootstrapping	tree from bayesian posterior distribution in found subgroups	*KRAS/NRAS/BRAF* alterations in left-sided	no new subgroup	0.2
SIDES	Subgroup identification based on differential effect search	R package 'SIDES'	differential effects search	Šidák multiplicity adjustment and independent validation	subgroups with interaction effects	none	-	1.9

Supplementary Table 1: Benchmark of OncoBird with other methods. Qualitative and quantitative comparison between OncoBird and previously published data-driven subgroup analysis methods, i.e. virtual twins (VT), model-based partitioning (MOB), an outcome-weighted method (OWE), causal random forests (CRF), policy learning (POL), GUIDE, PRISM and SIDES.

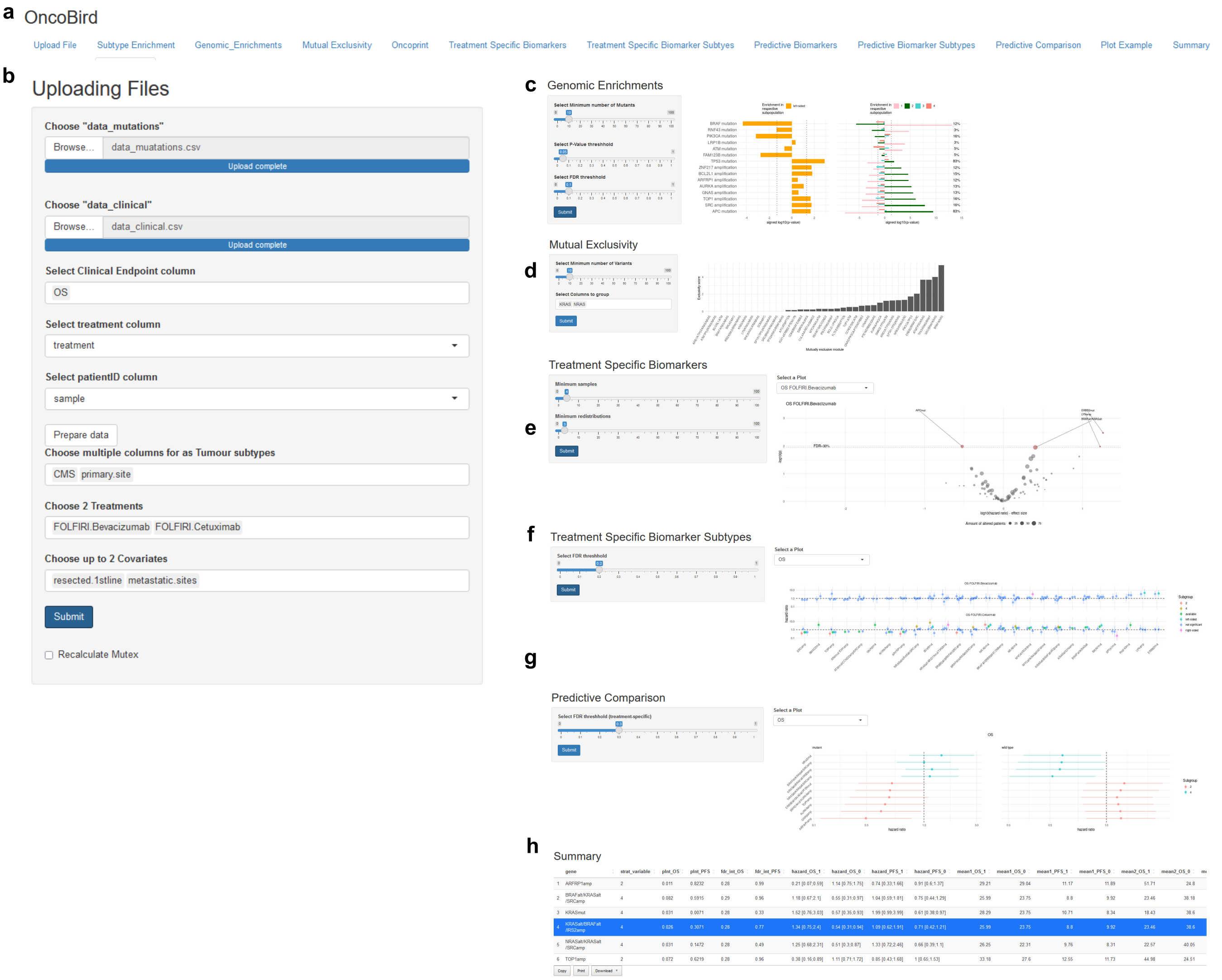

Supplementary Figure 1: OncoBird dockerised Shiny application. (**a**) Each tab corresponds to one analysis step, for example (**b**) the user interface for data input. Then, results are generated for (**c**) enrichment of genetic alterations in tumour subtypes, (**d**) mutual exclusivity, treatment-specific biomarkers in (**e**) all tumours and (**f**) in tumour subtypes. Also, results for predictive biomarkers using (**g**) interaction tests and (**h**) the table summary.

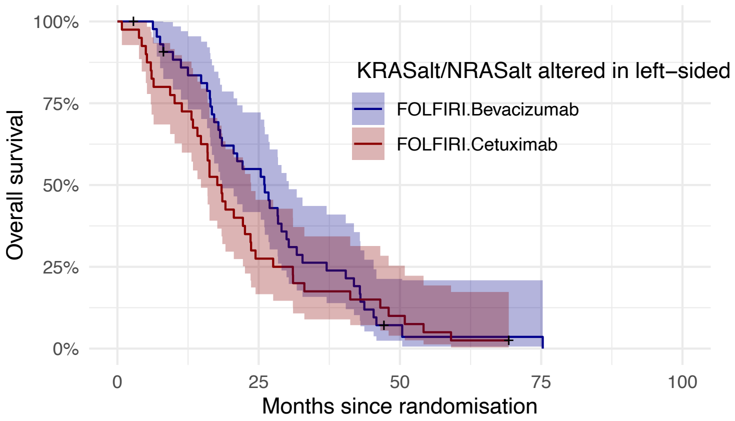

Supplementary Figure 2: Predictive biomarkers in metastatic colorectal cancer. Kaplan-Meier plot including 95% confidence intervals (CI) showing left-sided tumours that are mutated in either *KRAS* or *NRAS* treated with either cetuximab or bevacizumab.

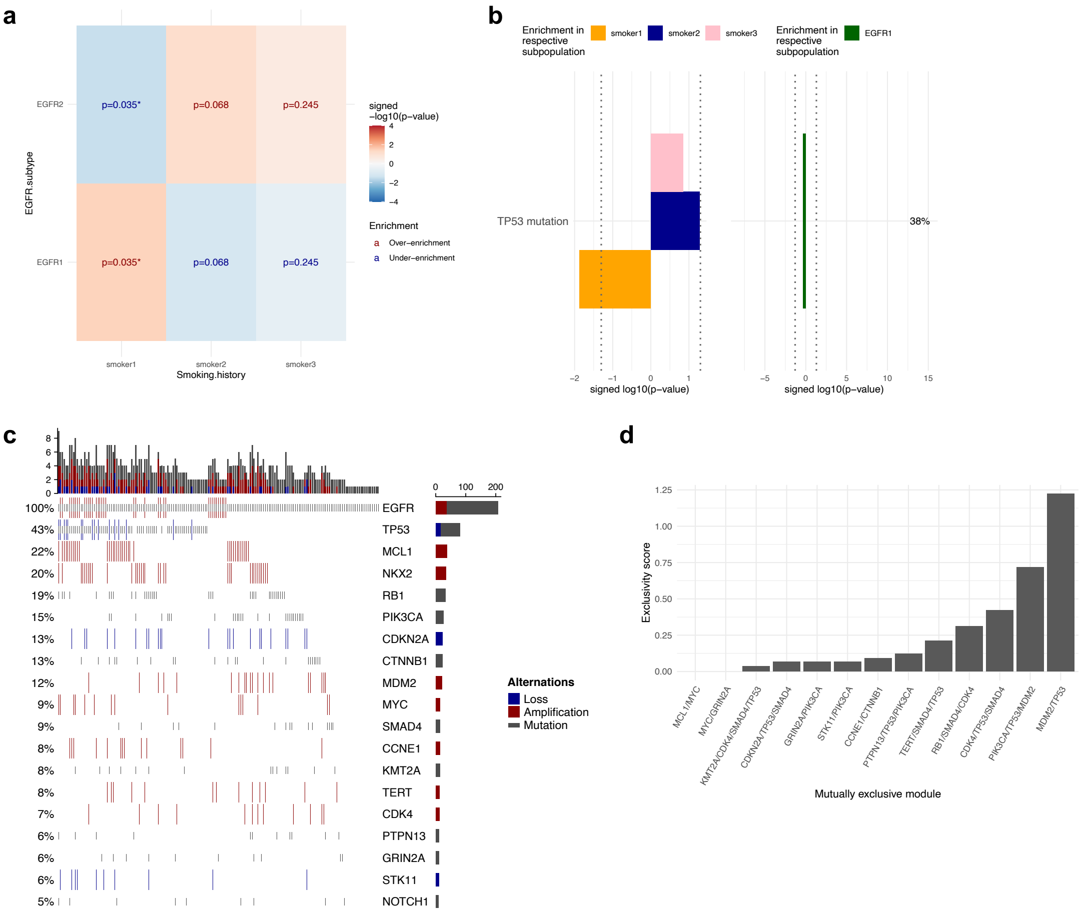

Supplementary Figure 3: The molecular landscape of non-small cell lung cancer. (**a**) Interactions between EGFR subtype and smoking status as tumour subtypes showing raw p-values derived from one-sided hypergeometric tests. (**b**) Altered cancer genes tested for enrichment in these tumour subtypes using one-sided hypergeometric tests showing raw p-values. (**c**) Oncoprint of 171 tumours, including mutations and copy number alterations detected in more than ten tumours. (**d**) The mutually exclusive alteration patterns were derived with the Mutex algorithm. Source data for the figure panels are provided as **Source Data** file.

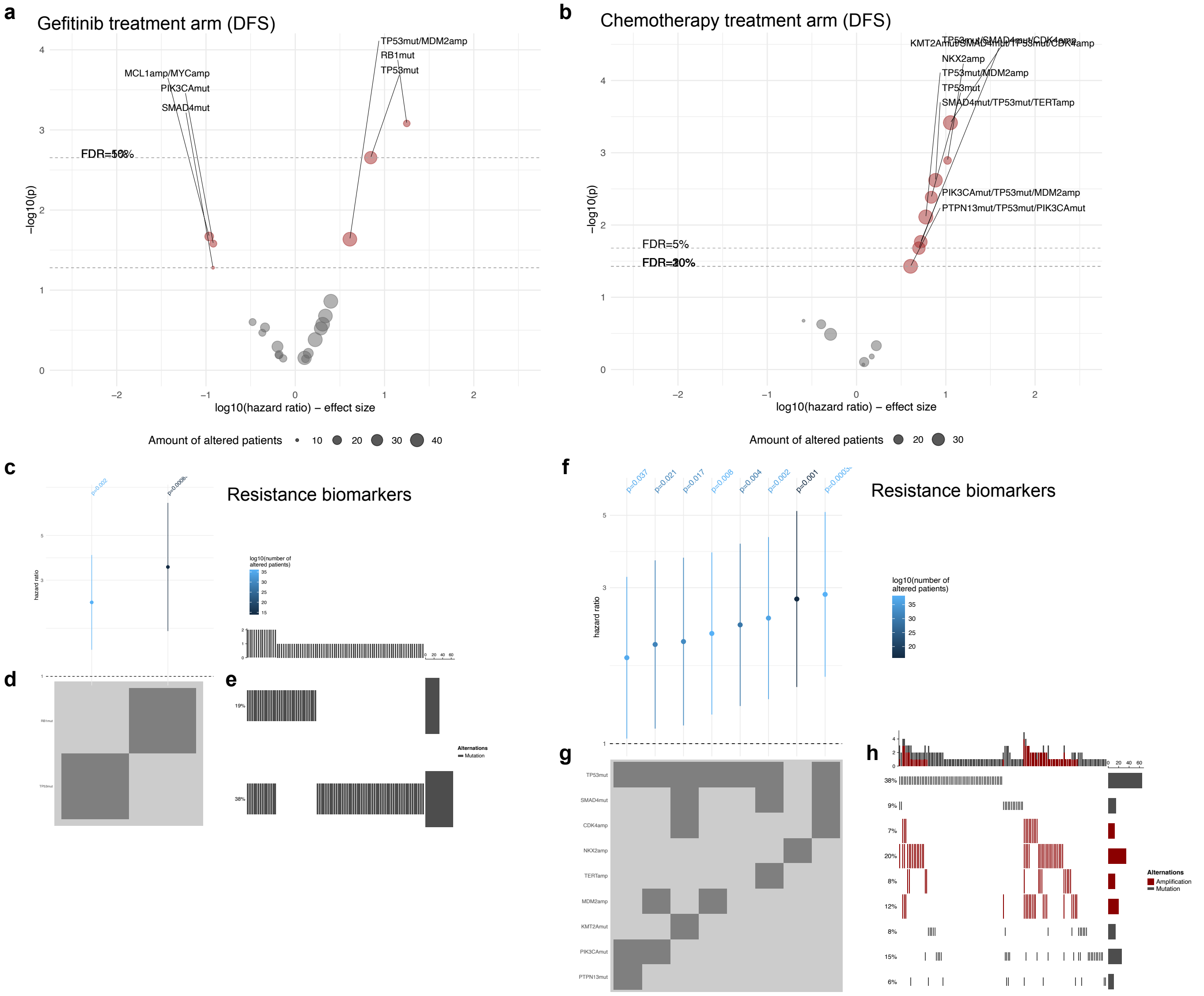

Supplementary Figure 4: Genetic biomarkers of DFS for gefitinib or chemotherapy. Prognostic value of mutually exclusive gene modules or single gene mutations for (**a**) gefitinib or (**b**) chemotherapy. Each point shows the effect of a particular group of alterations summarised by its hazard ratio derived by the Cox regression models and its raw p-value derived by a Wald test. We focus on (**c**) resistance biomarkers of gefitinib with $\mathrm{FDR}_{gef/che} < 0.1$, showing their hazard ratios and 95% confidence intervals (CI), (**d**) the composition of mutually exclusive gene modules indicated by dark grey colour, and (**e**) an oncoprint highlighting mutational frequencies and patterns. In like manner, (**f**) chemotherapy resistance biomarkers with their hazard ratios and 95% CIs and their (**g**) composition are shown with (**h**) their oncoprint. A **Source Data** file is provided, which contains the source data for the figure panels and the sample sizes of the conducted statistical tests.

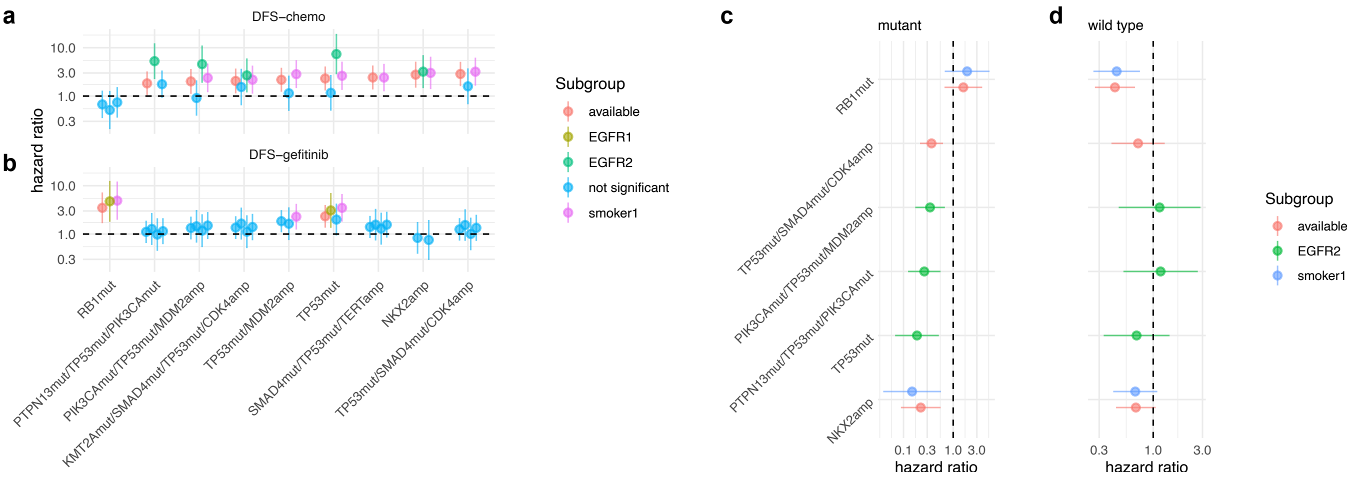

Supplementary Figure 5: Identification of subtype-specific predictive biomarkers for gefitinib or chemotherapy. Subtype-specific genetic biomarkers for DFS of (**a**) chemotherapy and (**b**) gefitinib using hazard ratios including 95% confidence intervals (CI) derived from single Cox regression models. Subtypes are defined by either the EGFR subtype, smoking status or unstratified (reference model). A **Source Data** file is provided, which contains the source data for the figure panels and the sample sizes of the conducted statistical tests. Overview of interaction biomarkers ($FDR_{int} < 0.2$) focusing on (**c**) mutant and (**d**) wild type tumours, using hazard ratios including 95% CIs derived from single Cox regression models fitted on DFS when comparing gefitinib or chemotherapy treatment. For the conducted statistical tests, the sample sizes are given in **Supplementary Data 2**. Source data for the figure panels are provided as **Source Data** file.

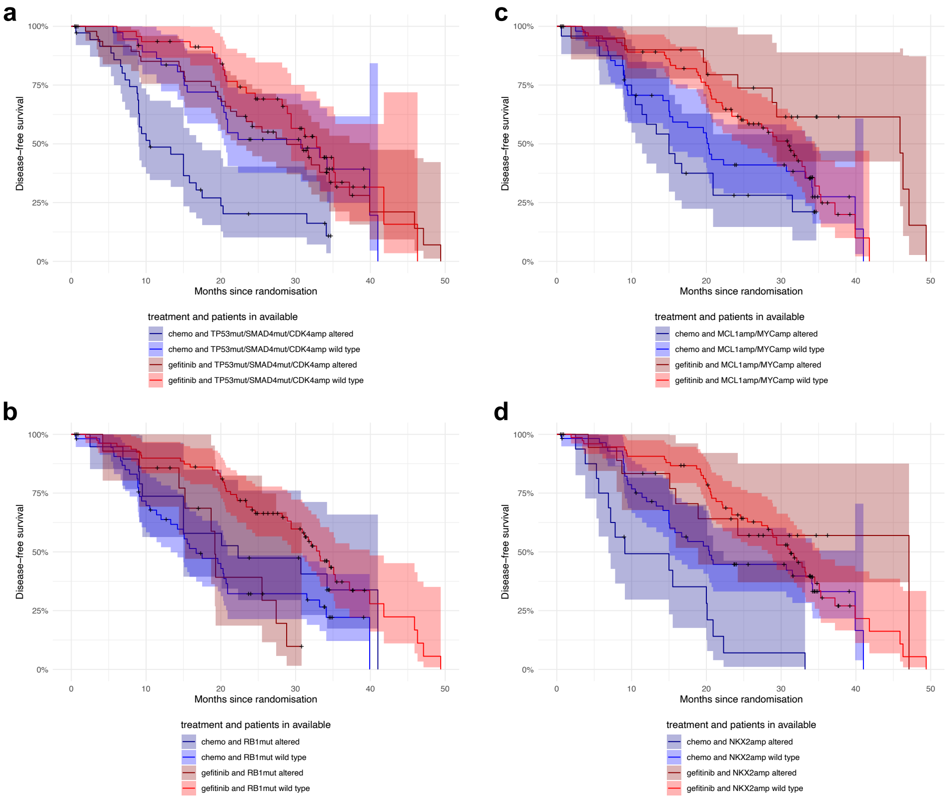

Supplementary Figure 6: Predictive biomarkers for gefitinib. Kaplan-Meier plots including 95% confidence intervals (CI) of genetic biomarkers for (**a**) mutually exclusive module consisting of mutations in *TP53*, *SMAD4* or *CDK4* amplifications, (**b**) *RB1* mutations, (**c**) amplifications in *MCL1* or *MYC*, and (**d**) *NKX2-1* amplifications across all patients in either the gefitinib or chemotherapy treatment arm.

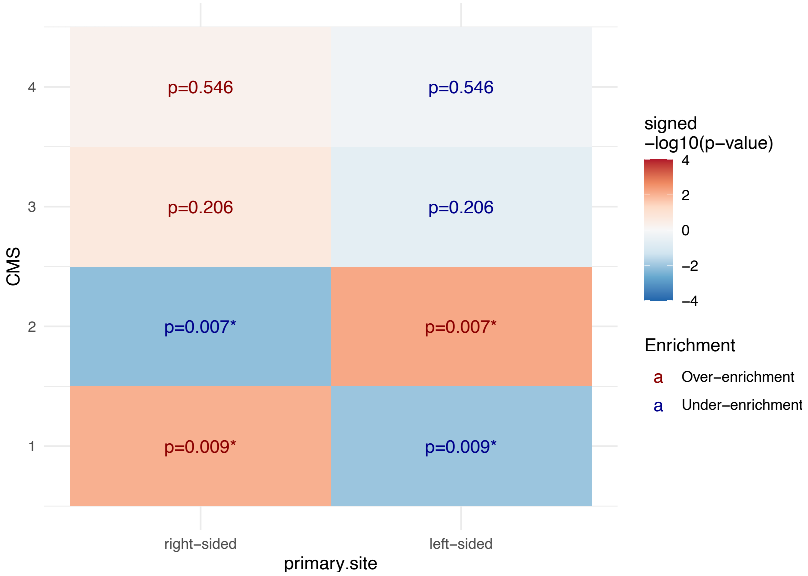

Supplementary Figure 7: Tumour subtypes in metastatic colorectal cancer. Interactions between tumour sidedness and CMS subtypes showing raw p-values derived from one-sided hypergeometric tests.

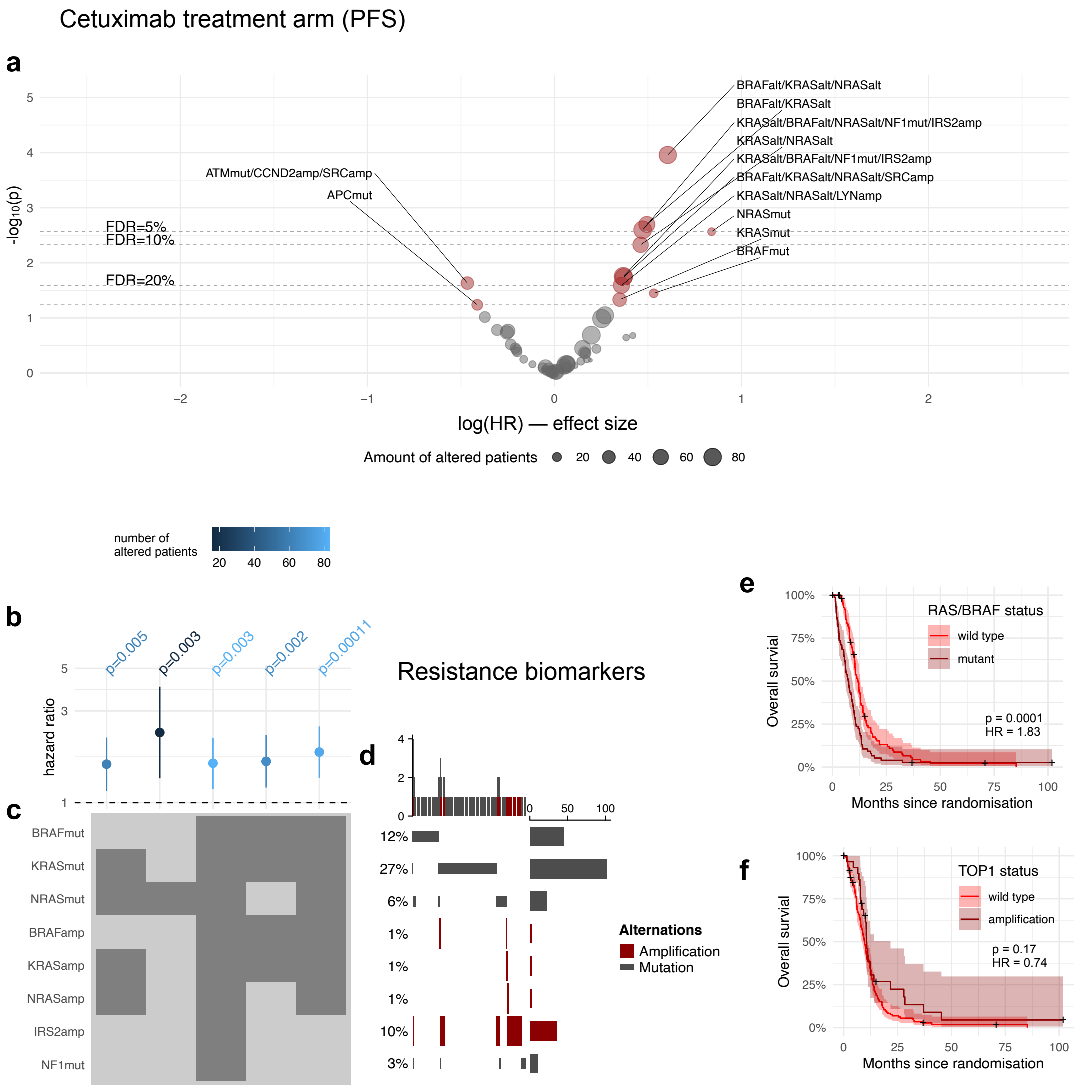

Supplementary Figure 8: Genetic biomarkers of PFS for cetuximab. (**a**) Prognostic value of mutually exclusive gene modules or single gene mutations for cetuximab. Each point shows the effect of a particular group of alterations summarised by its hazard ratio derived by the Cox regression models and its raw p-value derived by a Wald test. We focus on (**b**) resistance biomarkers of FOLFIRI plus cetuximab with $FDR_{cet} < 0.1$, showing their hazard ratios and 95% confidence intervals (CI), (**c**) the composition of mutually exclusive gene modules indicated by dark grey colour, and (**d**) an oncoprint highlighting mutational frequencies and patterns. Kaplan-Meier plot including 95% CIs and summary statistics of the Cox regression models for (**e**) *RAS* or *BRAF* mutations and (**f**) *TOP1* amplifications. No sensitivity biomarkers were found with $FDR_{cet} < 0.1$. A **Source Data** file is provided, which contains the source data for the figure panels and the sample sizes of the conducted statistical tests.

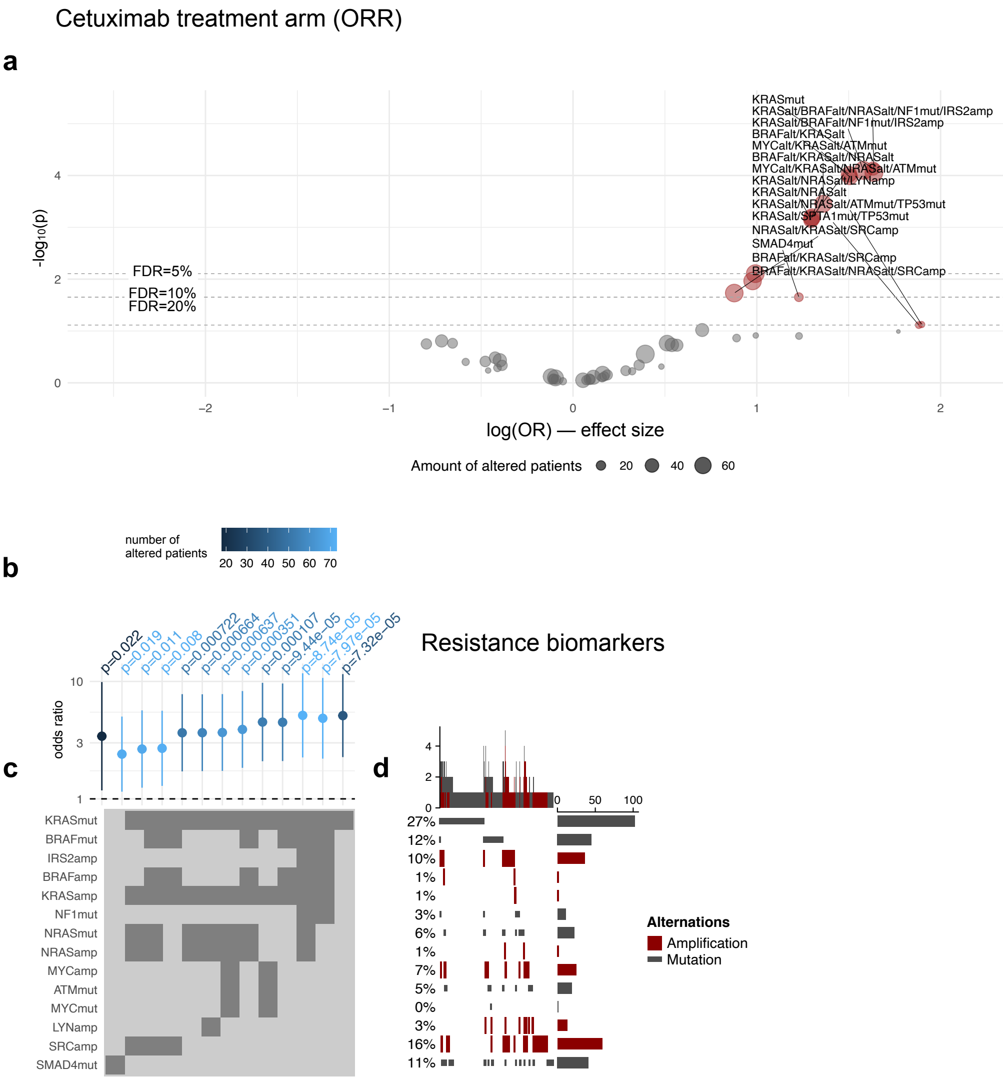

Supplementary Figure 9: Genetic biomarkers of ORR for cetuximab. (**a**) Prognostic value of mutually exclusive gene modules or single gene mutations for cetuximab. Each point shows the effect of a particular group of alterations summarised by its odds ratio derived by the logistic regression models and its raw p-value derived by a Wald test. We focus on (**b**) resistance biomarkers of FOLFIRI plus cetuximab with $\mathrm{FDR}_{cet} < 0.1$ showing their odds ratios and 95% confidence intervals (CI), (**c**) the composition of mutually exclusive gene modules indicated by dark grey colour, and (**d**) an oncoprint highlighting mutational frequencies and patterns. No sensitivity biomarkers were found with $\mathrm{FDR}_{cet} < 0.1$. A **Source Data** file is provided, which contains the source data for the figure panels and the sample sizes of the conducted statistical tests.

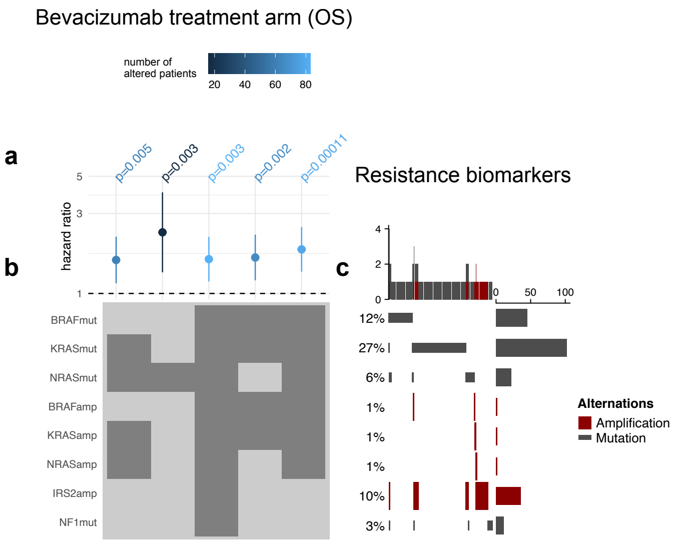

Supplementary Figure 10: Genetic biomarkers of OS for bevacizumab. We focus on (**a**) resistance biomarkers of FOLFIRI plus bevacizumab with $\text{FDR}_{bev} < 0.3$, showing their hazard ratios and 95% confidence intervals (CI), (**b**) the composition of mutually exclusive gene modules indicated by dark grey colour, and (**c**) an oncoprint highlighting mutational frequencies and patterns. Apart from *APC* mutations, no further sensitivity biomarkers were found with $\text{FDR}_{bev} < 0.3$. A **Source Data** file is provided, which contains the source data for the figure panels and the sample sizes of the conducted statistical tests.

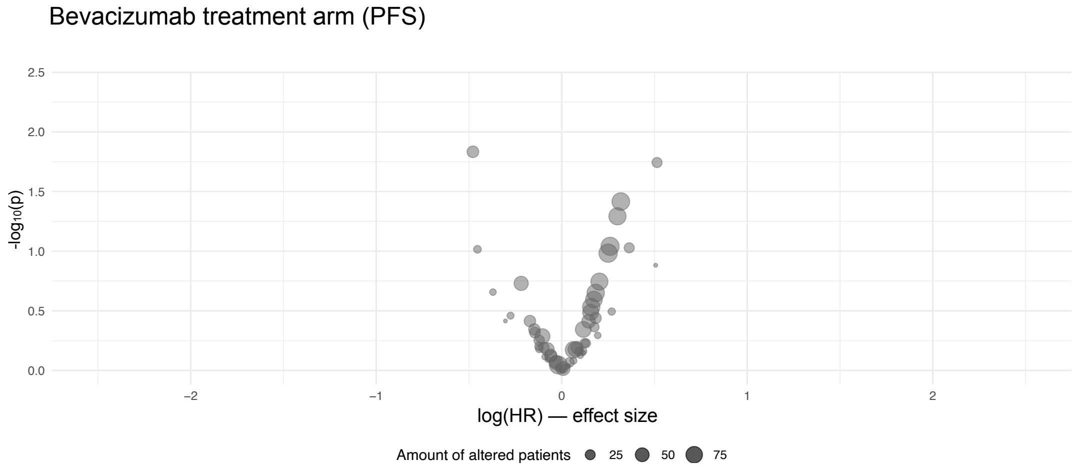

Supplementary Figure 11: Genetic biomarkers of PFS for bevacizumab. No prognostic value of mutually exclusive gene modules or single gene mutations for bevacizumab. Each point shows the effect of a particular group of alterations summarised by its hazard ratio derived by the Cox regression models and its raw p-value derived by a Wald test. A **Source Data** file is provided, which contains the source data for the figure panels and the sample sizes of the conducted statistical tests.

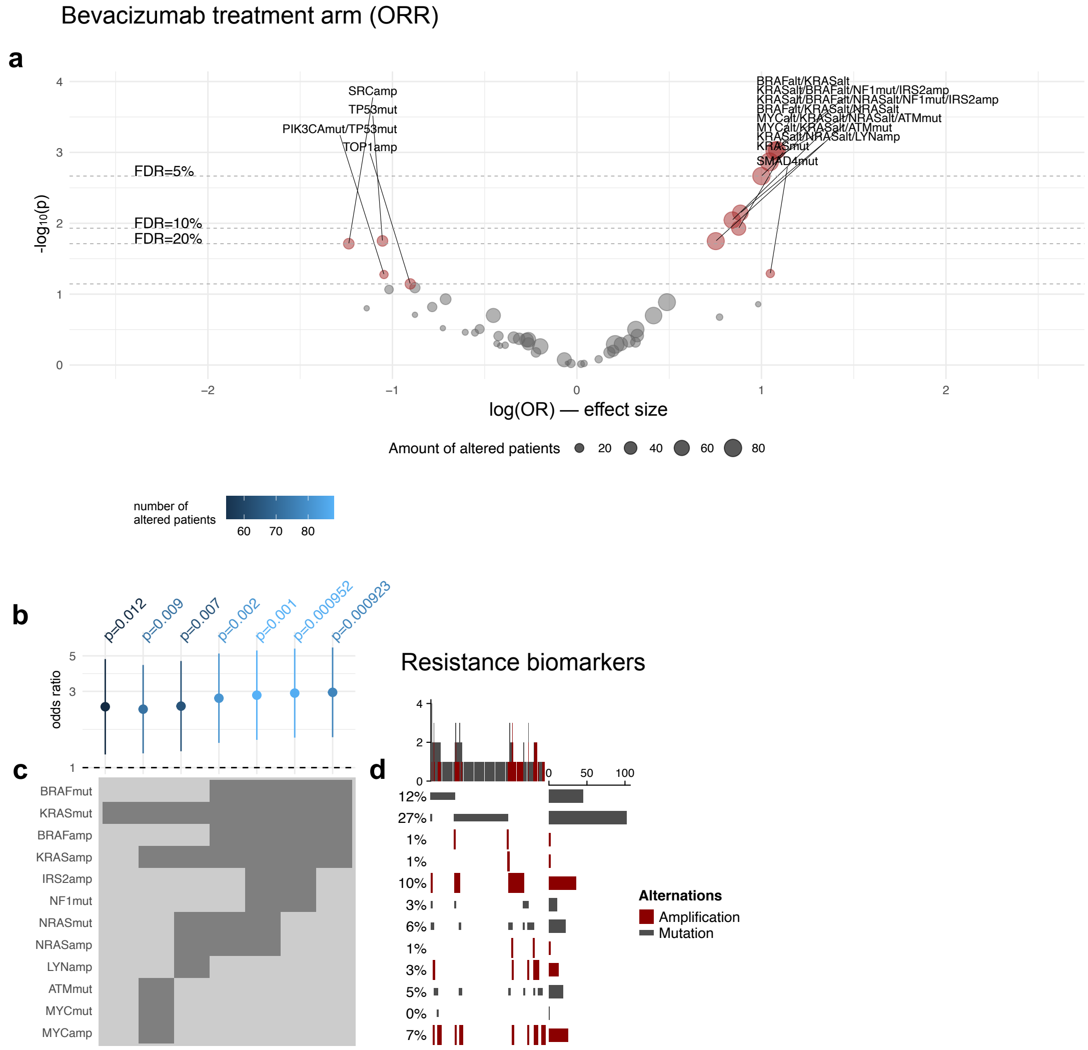

Supplementary Figure 12: Genetic biomarkers of ORR for bevacizumab. (**a**) Prognostic value of mutually exclusive gene modules or single gene mutations for bevacizumab. Each point shows the effect of a particular group of alterations summarised by its odds ratio derived by the logistic regression models and its raw p-value derived by a Wald test. We focus on (**b**) resistance biomarkers of FOLFIRI plus bevacizumab with $FDR_{bev} < 0.1$, showing their hazard ratios and 95% confidence intervals (CI), (**c**) the composition of mutually exclusive gene modules indicated by dark grey colour, and (**d**) an oncoprint highlighting mutational frequencies and patterns. No sensitivity biomarkers were found with $FDR_{bev} < 0.1$. A **Source Data** file is provided, which contains the source data for the figure panels and the sample sizes of the conducted statistical tests.

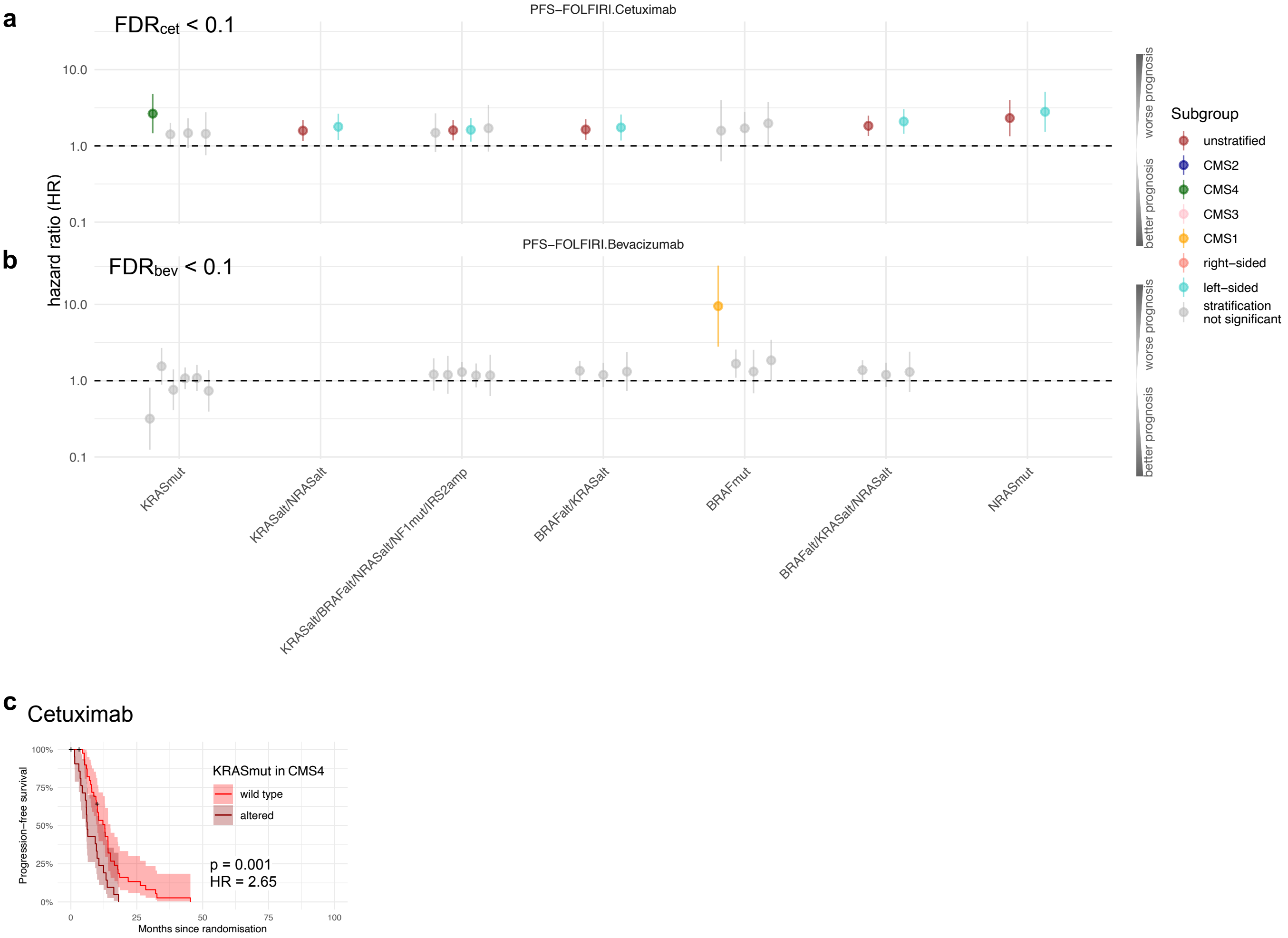

Supplementary Figure 13: Identification of subtype-specific biomarkers for FOLFIRI plus cetuximab or bevacizumab in terms of PFS. Subtype-specific prognostic value of genetic markers for PFS of (**a**) cetuximab and (**b**) bevacizumab using hazard ratios including 95% confidence intervals (CI) derived from single Cox regression models. Subtypes are defined by either the primary tumour side, CMS or unstratified (reference model). (**c**) Kaplan-Meier plot including 95% CIs, hazard ratios and raw p-values derived by Wald tests from the Cox regression models showing PFS of CMS4 tumours stratified by *KRAS* mutations treated with cetuximab. A **Source Data** file is provided, which contains the source data for the figure panels and the sample sizes of the conducted statistical tests.

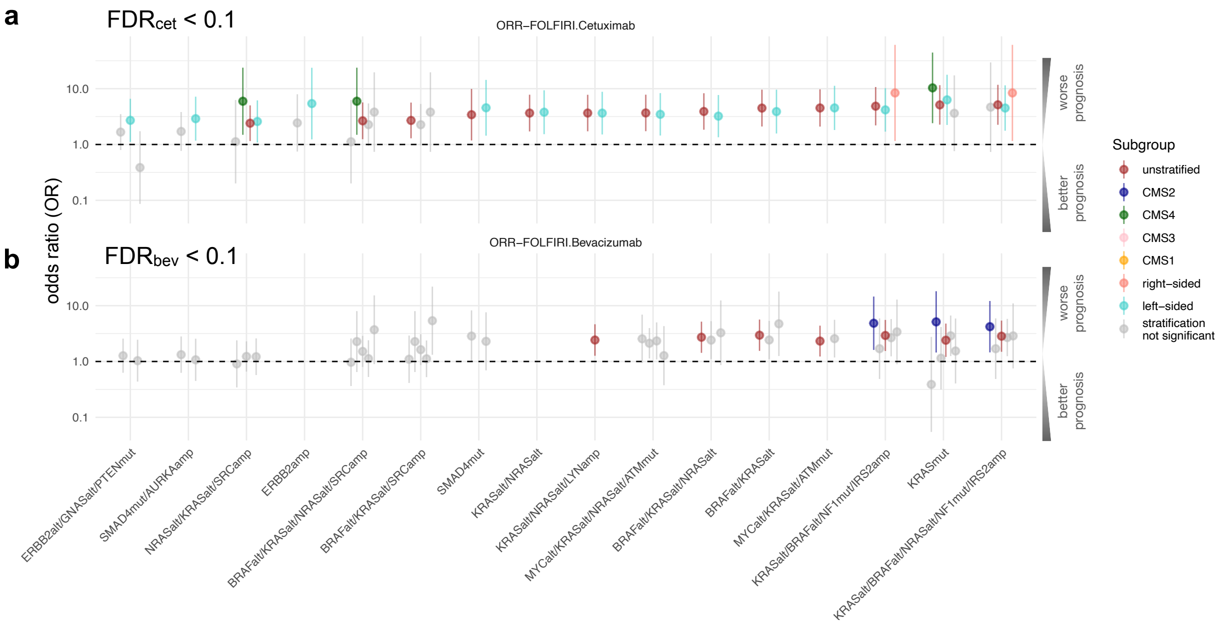

Supplementary Figure 14: Identification of subtype-specific biomarkers for FOLFIRI plus cetuximab or bevacizumab in terms of ORR. Subtype-specific prognostic value of biomarkers for ORR of (**a**) cetuximab and (**b**) bevacizumab using odds ratios including 95% confidence intervals (CI) derived from single logistic regression models. Subtypes are defined by either the primary tumour side, CMS or unstratified (reference model). A **Source Data** file is provided, which contains the source data for the figure panels and the sample sizes of the conducted statistical tests.

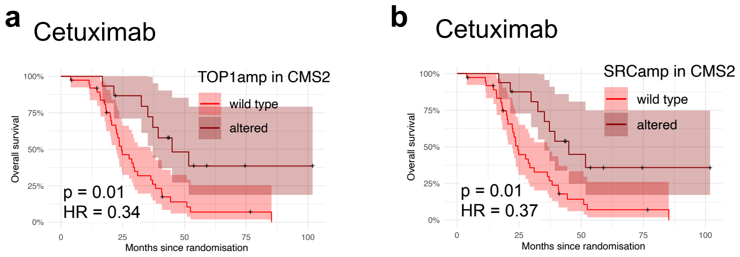

Supplementary Figure 15: Amplifications in chr20q in tumours treated with cetuximab. Kaplan-Meier plots including 95% CIs, hazard ratios and raw p-values derived by Wald tests from the Cox regression models of subtype-specific prognostic biomarkers for OS and cetuximab showing (**a**) *TOP1* amplifications or *SRC* amplifications in CMS2, respectively.

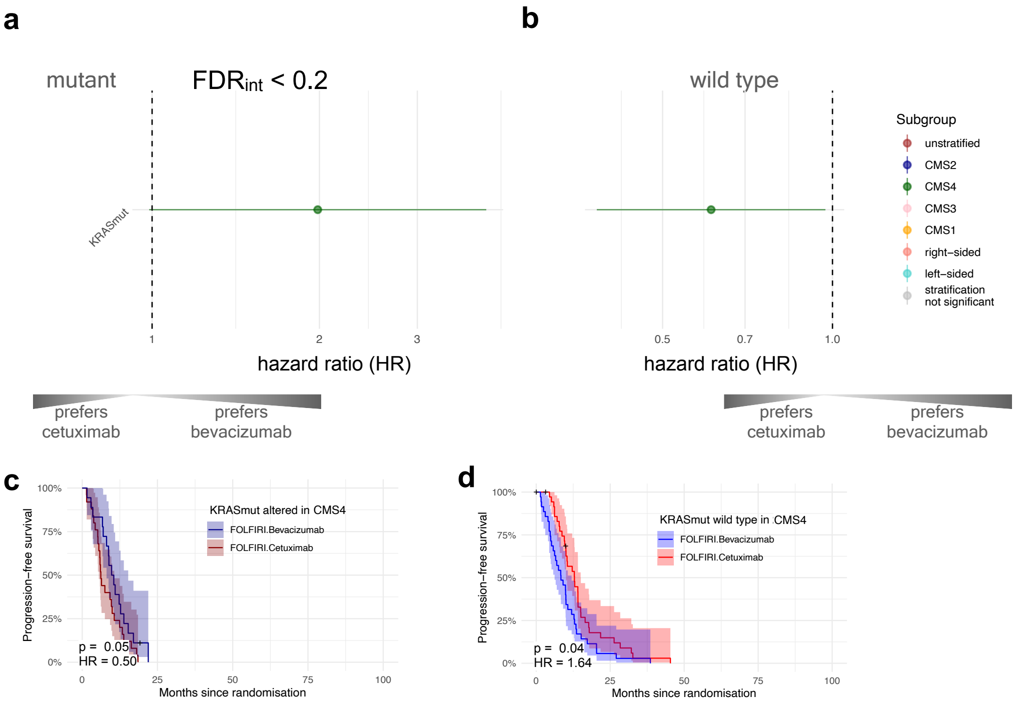

Supplementary Figure 16: Predictive interaction biomarkers in the context of tumour subtypes in terms of PFS. Overview of interaction biomarkers focusing on (**a**) mutant and (**b**) wild type populations when comparing cetuximab and bevacizumab with an interaction $\text{FDR}_{int} < 0.2$ using hazard ratios including 95% confidence intervals (CI) derived from single Cox regression models fitted on PFS. For the conducted statistical tests, the sample sizes are given in **Supplementary Data 3**. Here exemplified, Kaplan-Meier plots including 95% CIs, hazard ratios and raw p-values derived by Wald tests from the Cox regression models comparing mutant and wild type cohorts of (**c**,**d**) *KRAS* mutations in CMS4. Source data for the figure panels are provided as **Source Data** file.
