# Supplementary material for: The Oncology Biomarker Discovery framework reveals cetuximab and bevacizumab response patterns in metastatic colorectal cancer
Source: Nat Commun. 2023 Sep 4;14:5391. doi: 10.1038/s41467-023-41011-4 (PMC10477267; doi:10.1038/s41467-023-41011-4)
Supplement: Supplementary file 4 — Description of Additional Supplementary Files [file 41467_2023_41011_MOESM4_ESM.docx]

**Description of additional Supplementary Files**

**Supplementary Data 1: Pseudocode of five functions that constitute the analysis steps performed by OncoBird.**

**Supplementary Data 2: OncoBird applied to the ADJUVANT clinical trial for non-small cell lung cancer (NSCLC) to discover response biomarkers for gefitinib.** Tabulated are eight subtype-specific biomarkers with predictive components with interaction FDR_int_ < 0.2 and treatment-specific differential prognosis FDR_gef/che_ < 0.1 in either readout (DFS or OS). We display the interaction p-value, FDR and hazard ratio for each mutant and wild type population. Furthermore, we display the p-value for the treatment comparison as well as median DFS and OS, and number of samples used in the statistical tests. Finally, we show the p-values and FDR for the treatment-specific tests and the corresponding hazard ratios. The hazard ratios are tabulated as HR < 1 if the mutant tumours display a better prognosis than the wild type tumours. For the treatment comparison, HR < 1 and HR > 1 correspond to benefit with gefitinib and chemotherapy, respectively. Finally, we show the corrections of the treatment effect p-value and its bias-corrected confidence intervals in terms of DFS.

**Supplementary Data 3: Landscape of predictive biomarkers in the FIRE-3 clinical trial.** Tabulated are 57 subtype-specific biomarkers with predictive components with interaction FDR_int_ < 0.6 and treatment-specific differential prognosis FDR_cet/bev_ < 0.1 in either readout (OS, PFS or ORR). Each row corresponds to one genetic alteration and its respective subtype which was tested for predictive effects. We display the interaction p-value, FDR and hazard/odds ratio for each mutant and wild type population. Furthermore, we display the p-value for the treatment comparison as well as median OS and PFS, ratio of ORR and number of samples used in the statistical tests. Finally, we show the p-values and FDR for the treatment-specific tests and the corresponding hazard/odds ratios. The hazard/odds ratios are tabulated as HR/OR < 1 if the mutant tumours display a better prognosis than the wild type tumours. For the treatment comparison, HR/OR < 1 and HR/OR > 1 correspond to benefit with cetuximab and bevacizumab, respectively.

**Supplementary Data 4: Significant biomarkers in the FIRE-3 clinical trial.** Tabulated are final five subtype-specific biomarkers with predictive components with interaction FDR_int_ < 0.2 and treatment-specific differential prognosis FDR_cet/bev_ < 0.1 in either readout (OS, PFS or ORR). We display the interaction p-value, FDR and hazard/odds ratio for each mutant and wild type population. Furthermore, we display the p-value for the treatment comparison as well as median OS and PFS, ratio of ORR and number of samples used in the statistical tests. Finally, we show the p-values and FDR for the treatment-specific tests and the corresponding hazard/odds ratios. The hazard/odds ratios are tabulated as HR/OR < 1 if the mutant tumours display a better prognosis than the wild type tumours. For the treatment comparison, HR/OR < 1 and HR/OR > 1 correspond to benefit with cetuximab and bevacizumab, respectively. Finally, we show the corrections of the treatment effect p-value and its bias-corrected confidence intervals in terms of OS.
