# Supplementary material for: The Oncology Biomarker Discovery framework reveals cetuximab and bevacizumab response patterns in metastatic colorectal cancer
Source: Nat Commun. 2023 Sep 4;14:5391. doi: 10.1038/s41467-023-41011-4 (PMC10477267; doi:10.1038/s41467-023-41011-4)
Supplement: Supplementary file 5 — Supplementary Data 1 [file 41467_2023_41011_MOESM5_ESM.pdf]

Input: *MUT* // $n \times m$ data frame containing n patients and m binary genetic alterations including single nucleotide variants ('SV'), amplifications ('AMP') an deletions ('DEL')
CLIN // $n \times k$ data frame containing n patients and k columns including clinical endpoints, a treatment indicator ('treatment'), subtypes ('subtypes') and additional covariates

Algorithm: OncoBird workflow

```
function GET-MUTATIONS-IN-SUBTYPES(MUT, CLIN)
    // Get enrichments of mutations in tumour subtypes
    scores.e ← list()                                   // initialise empty scores list for enrichments
    q ← LENGTH(CLIN['subtypes'])                         // count how many subtypes in column
    for j = 1 to m do
        for s = 1 to q do
            score ← HYPERGEOMETRIC-TEST(j, s, MUT, CLIN) // test for enrichment or depletion
            scores.e ← APPEND(score, scores.e)
        end for
    end for
    scores.e.adjusted ← BENJAMINI-HOCHBERG-CORRECTION(scores.e) // multiplicity adjustment
        for all scores
    return scores.e.adjusted
end function

function GET-MUTATIONS-MODULES(MUT)
    // Call mutually exclusive modules of somatic mutations
    MUT.modules ← MUTEX-ALGORITHM(MUT)
    return MUT.modules
end function

function GET-TREATMENT-SPECIFIC-BIOMARKERS(MUT, CLIN)
    // Model outcome for each treatment arm and correct for multiplicity
    scores.tsb ← list()           // initialise empty scores list for treatment-specific biomarkers
    T ← LENGTH(CLIN['treatment'])                         // count how many treatments in column
    q ← LENGTH(CLIN['subtypes'])                          // count how many subtypes in column
    for t = 1 to T do
        for j = 1 to m do
            score.t ← TREATMENT-SPECFIC-MODEL-FIT(t, j, MUT, CLIN)  // subtype-independent
                models
            scores.tsb ← APPEND(score.t, scores.tsb)
        end for
        scores.tsb.adjusted ← BENJAMINI-HOCHBERG-CORRECTION(t, scores.tsb)     // multiplicity
            adjustment for each treatment arm
        for s = 1 to q do
            for j = 1 to m do
                score.t ← TREATMENT-SPECFIC-MODEL-FIT(t, j, s, MUT, CLIN) // subtype-specific
                    models
                scores.tsb ← APPEND(score.t, scores.tsb)
            end for
        end for
        scores.tsb.adjusted ← BENJAMINI-HOCHBERG-CORRECTION(t, scores.tsb)     // multiplicity
            adjustment for each treatment arm across subtypes
    end for
    return scores.tsb.adjusted
end function

function GET-PREDICTIVE-BIOMARKERS(MUT, CLIN)
    // Model outcome with treatment interactions and correct for multiplicity
```

```
    scores.pd ← list()                                   // initialise empty scores list for predictive biomarkers
    q ← LENGTH(CLIN['subtypes'])                         // count how many subtypes in column
    for j = 1 to m do
        score.pb ← INTERACTION-MODEL-FIT(j, MUT, CLIN)   // subtype-independent models
        scores.pb ← APPEND(score.pb, scores.pb)
    end for
    scores.pb.adjusted ← BENJAMINI-HOCHBERG-CORRECTION(scores.pb)   // multiplicity
        adjustment for all scores
    for s = 1 to q do
        for j = 1 to m do
            score.pb ← INTERACTION-MODEL-FIT(j, s, MUT, CLIN)   // subtype-specific models
            scores.pb ← APPEND(score.pb, scores.pb)
        end for
    end for
    scores.pb.adjusted ← BENJAMINI-HOCHBERG-CORRECTION(scores.pb)   // multiplicity
        adjustment across subtypes
    return scores.pb.adjusted
end function

function GET-CONDITIONAL-AVERAGE-TREATMENT-EFFECT(MUT, CLIN, scores.pb.adjusted, scores.tsb.adjusted,
        fdr.pb, fdr.tsb)
        // Get subgroups from biomarkers with predictive components and estimate conditional aver-
        age treatment effects with resampling
    scores.cate ← list()                                 // initialise empty scores list for treatment effects
    subgroup ← GET-PREDICTIVE-GENE-IN-SUBTYPE(
            scores.pb.adjusted < fdr.pd,
            scores.tsb.adjusted < fdr.tsb)   // get biomarkers and subtypes with treatment-specific and
            predictive component
    scores.cate ← GET-CATE(subgroup, MUT, CLIN)          // get unadjusted treatment effect in
            subgroup
    scores.cate.adjusted ← PERMUTATION-CORRECTION(scores.cate)   // adjust treatment effect
            p-values
    scores.cate.adjusted.CIs ← BOOTSTRAP-CORRECTION(scores.cate)   // adjust treatment effect
            confidence intervals
    return scores.cate.adjusted, scores.cate.adjusted.CIs
end function


// Run OncoBird

GET-MUTATIONS-IN-SUBTYPES(MUT, CLIN)
GET-MUTATIONS-MODULES(MUT)
GET-TREATMENT-SPECIFIC-BIOMARKERS(MUT, CLIN)
GET-PREDICTIVE-BIOMARKERS(MUT, CLIN)
GET-CONDITIONAL-AVERAGE-TREATMENT-EFFECT(MUT, CLIN,
        GET-PREDICTIVE-BIOMARKERS(),
        GET-TREATMENT-SPECIFIC-BIOMARKERS(),
        fdr.pd,
        fdr.tsb)
```
